# ESC consensus statement on stroke risk management in carotid atherosclerotic disease: 10 key points

**DOI:** 10.1093/eurheartj/ehaf614

**Published:** 2025-09-26

**Authors:** Piotr Musialek, Iris Q Grunwald, Adnan H Siddiqui

**Affiliations:** Department of Cardiac and Vascular Diseases, Jagiellonian University, Krakow, Poland; St. John Paul II Hospital, ul. Pradnicka 80, 31-202 Krakow, Poland; Neuroradiology, University of Dundee, Dundee, UK; Image Guided Therapy Research Facility (IGTRF), University of Dundee, Dundee, UK; Department of Clinical Radiology and Imaging, NHS Tayside Ninewells Hospital, Dundee, UK; Department of Neurosurgery, Gates Vascular Institute at Kaleida Health, Buffalo, NY, USA; Canon Stroke and Vascular Research Center, University at Buffalo, Buffalo, NY, USA; Department of Radiology, Jacobs School of Medicine and Biomedical Sciences, University at Buffalo, Buffalo, NY, USA; Department of Neurosurgery, Jacobs School of Medicine and Biomedical Sciences, University at Buffalo, Buffalo, NY, USA

Despite unquestionable progress in pharmacologic therapies, carotid atherosclerotic disease (CarAD) remains an important cause of stroke, often disabling or fatal.^1,2^ Carotid stenosis is also a marker of overall cardiovascular risk.^1,3^ Advancements in imaging and pharmacology, progress in acute stroke management, and evidence from recent clinical trials and registries have markedly expanded the knowledge base for clinical decisions in CarAD.^1,3^

Medical specialties central to managing CarAD patients include neurology, radiology, stroke medicine, cardiology, angiology, ophthalmology, vascular surgery, neuroradiology, and neurosurgery. CarAD management guidelines from the different professional societies may vary in methodology and perspective, posing a challenge to consistence of patient care.^1^

To develop an effective consensus update on the contemporary management of CarAD, European Society of Cardiology (ESC) set up a multispecialty panel with experts from Europe and the USA. A diverse author group, including key opinion leaders of the different non-interventional and interventional specialties, enabled—along with a patient’s interests representative—a balanced approach to creating a comprehensive knowledge base for contemporary clinical decisions. The 10 key points of the document^1^ are summarized below.

## Carotid-related strokes can be largely prevented

Atherosclerotic carotid stenosis is a major, modifiable risk factor of ischaemic stroke.^1,3^ Stroke prevention strategies include screening, risk factor control, optimal medical therapy, and carotid revascularization for patients at increased stroke risk. Preventing carotid-related strokes is paramount, as once a disabling stroke occurs no treatment can currently reverse the cerebral tissue loss.

## Stroke risk dictates management

**Figure 1:**
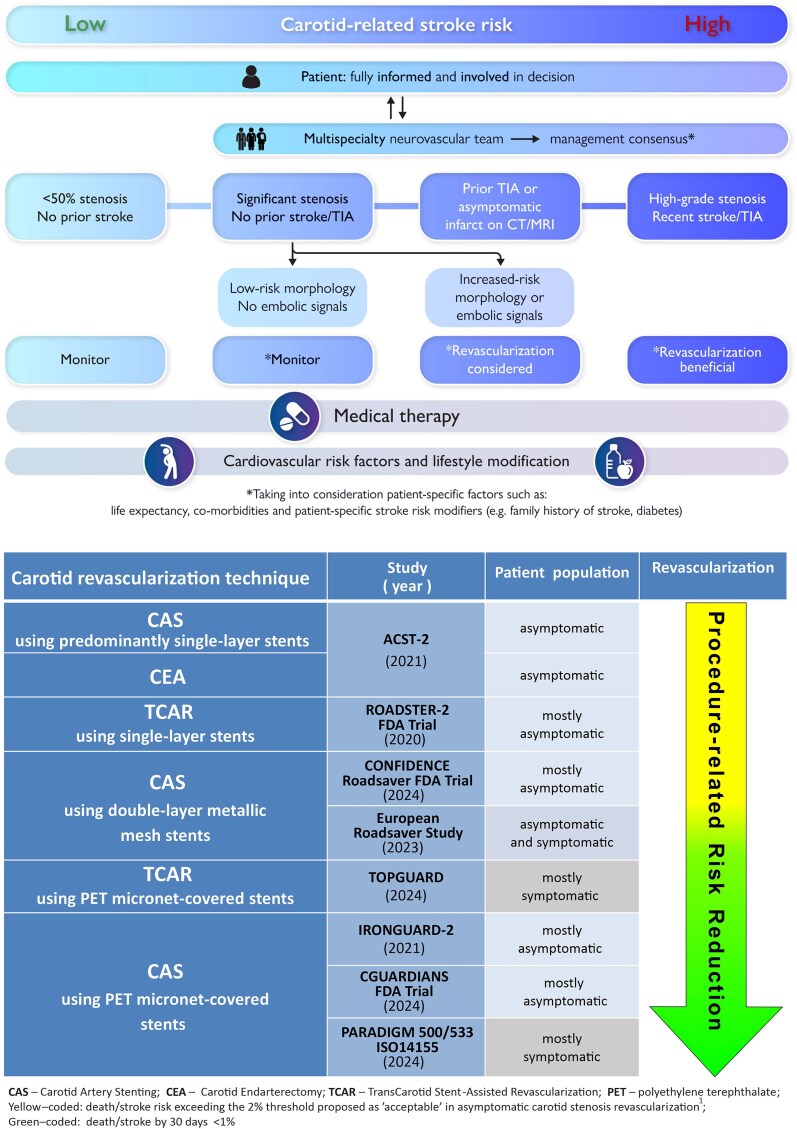
Stroke risk stratification determines management of carotid stenosis (top^1^) and carotid revascularization risk reduction seen with technology advancements (bottom). The top graphic illustrates patient management in the context of the gradient of stroke risk with atherosclerotic carotid disease, from low to high. ‘Significant’ stenosis is typically defined as ≥50% reduction of the carotid artery luminal diameter, but plaque morphology emerges as an important determinant of stroke risk. Patients with increased stroke risk should be evaluated for revascularization on top of cardiovascular risk factors and lifestyle modification and optimized medical therapy. The decision on performing vs deferring revascularization should ideally be based on a multidisciplinary (Neurovascular Team) consensus statement. To assist the patient in their decision, the Neurovascular Team may also advise a preferred revascularization mode (according to patient-specific factors and local expertise). The patient, holding a central position in the decision process regarding their care, requires full information about disease-related stroke risk and treatment options, including risks associated with the different treatments and their advantages. The graphic in the bottom presents the key carotid revascularization studies of the last 5 years, grouped according to the revascularization technique used and gradient in risk of periprocedural stroke or death

There is a gradient of stroke risk with carotid stenosis^1^ (*Figure 1*, top). Patients at increased stroke risk should be evaluated for revascularization on top of risk factor modification and maximized medical therapy.^1,3^ Validated risk prediction scores (analogous to the CHA_2_DS_2_-VASc scale or mCARS scale for patients with atrial fibrillation) are needed for patients with CarAD.^1,3^ Key risk factors for CarAD include diabetes, hypertension, a history of cardiovascular disease, and dyslipidaemia.^1^ Selective screening for CarAD can enhance risk factor management and optimize medical therapy.^1,3,4^ Targeted screening programs in well-defined populations could provide significant public health benefits.^1,4^

## Multidisciplinary neurovascular team approach

The management of vascular diseases—such as coronary artery disease, valvular heart disease, and peripheral arterial disease—has increasingly become a multispecialty task. Similarly, the treatment of acute stroke has evolved into a collaborative, multidisciplinary approach. The Neurovascular Team concept,^1^ modelled after the Heart Team, brings together diverse specialties to deliver personalized, stratified patient care. By integrating advanced imaging and risk assessment, the team ensures that treatment decisions are tailored to the patient needs.^1^ A multidisciplinary consensus helps translate messages from different applicable guidelines to the individual patient care. The collaborative approach can weigh up the advantages and disadvantages of medical therapy alone—carotid stenting or carotid surgery—and provide consistent advice to the patient^1^ (*Figure 1*, top).

## Patient involvement in decisions regarding their care

The patient, holding a central position in the decision process regarding their care^1^ (*Figure 1*, top) requires full information about disease-related stroke risk and treatment options, including risks associated with the different treatments and their advantages.^1^ The viable treatment options should be discussed with the patient, thereby allowing fully informed decision-making. Involving the patient (and, with consent, their caregivers or family) in the decision-making process may also help with long-term adherence to medical therapy. Patient preferences, including the mode of revascularization (if indicated), should be respected.^1^

## Plaque composition and morphology provide insight into stroke risk beyond the traditional metric of intraluminal stenosis

Landmark trials established the benefit of carotid endarterectomy and carotid artery stenting for patients with significant luminal stenosis, leading to a widespread reliance on this single metric which oversimplifies the complex and dynamic pathology of the disease process occurring in the wall of the carotid artery.^1,3,5^ Emerging evidence suggests that plaque morphology and composition are important determinants of stroke risk and should be integrated into contemporary risk stratification and clinical decision-making^1^ (*Figure 1*, top). The high-risk plaque features associated with an increased likelihood of stroke include: (i) structural changes—increasing plaque volume/progressive stenosis, intraluminal calcific nodules, ulcerations, endothelial erosions, a ruptured fibrous cap; (ii) composition markers—echolucency, neovascularization, intra-plaque haemorrhage, lipid-rich necrotic core, and intraluminal thrombus; and (iii) inflammatory factors.^1^ Recent large-scale studies confirm that plaque composition and morphology are at least as relevant as stenosis severity in determining stroke risk.^5^ Patient-level factors like a family history of stroke, diabetes, asymptomatic cerebral embolism, and reduced cerebrovascular reserve further influence stroke risk.^1^

## Patients with acute carotid-related stroke should undergo emergency recanalization

Acute carotid-related strokes, including tandem lesions (extracranial carotid and intracranial occlusions), require immediate endovascular stroke treatment, akin to other cerebral large-vessel occlusions. Carotid-related transient ischaemic attacks demand urgent addressing of the underlying cause by performing urgent revascularization, as delaying intervention can result in preventable brain damage. The ‘wait-and-see’ strategy in such cases is detrimental, given the often progressive cerebral injury, substantial-risk of recurrent stroke and the fact that a substantial proportion of stroke victims would prefer death to their life after stroke.^1,3^

## Carotid revascularization in patients at increased stroke risk

Some observational data suggest that stroke risk associated with CarAD may have declined in recent years, likely due to advances in medical management. However, contemporary acute carotid-related stroke presenters continue to include those on maximized pharmacologic therapy.^2^ Today, there is no randomized evidence to demonstrate the efficacy of pharmacologic therapy alone in reducing carotid-related stroke risk, nor is there any proof that medical management alone is sufficient for stroke prevention in CarAD.^1,3^ Many patients with CarAD remain at elevated stroke risk despite triple medical therapy (antiplatelet agents, statins, and antihypertensive medication). In such cases, low-risk, well-executed carotid revascularization can provide significant benefit. Advances in carotid interventions (such as local anaesthesia and intraoperative quality control via completion imaging in carotid endarterectomy and carotid artery stenting with improved cerebral protection and anti-embolic stents) have significantly improved outcomes^6,7^ (*Figure 1*, bottom). Long-term data indicate that carotid revascularization performed alongside medical therapy may offer durable protection against stroke.^1^

## Contemporary treatment decisions should be guided by contemporary evidence

Evidence-based medicine is not restricted to randomized controlled trials and meta-analyses; it involves tracking down the best available external evidence with which to answer the clinical question.^8^ The practice of evidence-based medicine means integrating individual clinical expertise with the best available contemporary external clinical evidence^8^ (*Figure 1*).

## The role of operator training, quality control and quality assurance

Competency in performing carotid interventions is essential for achieving optimal patient outcomes. A direct relationship exists between procedural volume and expertise, making structured training vital. Training on high fidelity simulators and advanced 3D printed models and perfused human cadaveric models is now highly realistic and recommended as part of credentialing in interventions in carotid-related stroke.^9^ Quality control, including procedural auditing, is important.

## Future directions

With low adverse event rates of contemporary carotid revascularization (*Figure 1*, bottom), appropriately powered large-scale randomized controlled trials are increasingly challenging to conduct. Studies powered for differences in cerebral embolism (a marker of procedural stroke risk) may play an increasing role in guiding clinical decisions.^1,10^ Well-maintained registries of management in all-comer (consecutive, unselected) patients provide valuable real-world data on procedural outcomes.^1^ The role of external data monitoring and external adjudication of adverse events to ensure quality of clinical studies and registries remains essential.^1^ Despite advancements in medical therapy, residual stroke risk persists, underscoring the continued need for competent carotid revascularization in selected patients.^1^ Future strategies will likely involve artificial intelligence applications to refine stroke risk stratification and treatment personalization.^1^

In conclusion, the ESC Trans-Atlantic Carotid Consensus Statement provides a multidisciplinary framework for optimizing stroke prevention and CarAD management. By integrating advanced imaging, optimized pharmacotherapy, competent interventions, and patient-centred multispecialty advice and care, this approach ensures tailored treatment decisions to maximize improved clinical outcomes for CarAD patients.

## References

[1] Musialek P, Bonati LH, Bulbulia R, Halliday A, Bock B, Capoccia L, et al Stroke risk management in carotid atherosclerotic disease: a clinical consensus statement of the ESC council on stroke and the ESC working group on aorta and peripheral vascular diseases. Cardiovasc Res 2025;121:13–43. 10.1093/cvr/cvad135 37632337

[2] Tekieli L, Dzierwa K, Grunwald IQ, Mazurek A, Urbanczyk-Zawadzka M, Wiewiorka L, et al Outcomes in acute carotid-related stroke eligible for mechanical reperfusion: SAFEGUARD-STROKE registry. J Cardiovasc Surg 2024;65:231–48. 10.23736/S0021-9509.24.13093-5 39007556

[3] Musialek P, Rosenfield K, Siddiqui AH, Grunwald IQ. Carotid stenosis and stroke: medicines, stents, surgery—“wait-and-see” or protect? Thromb Haemost 2024;124:815–27. 10.1055/a-1952-1159 36170885 PMC11349427

[4] Paraskevas KI, Musialek P, Lip GYH, Chaturvedi S. Selective screening for asymptomatic carotid artery stenosis: an appraisal of the 2024 European Society of Cardiology (ESC) guidelines position. Am J Med 2025;138:209–11. 10.1016/j.amjmed.2024.10.033 39547462

[5] Kamtchum-Tatuene J, Noubiap JJ, Wilman AH, Saqqur M, Shuaib A, Jickling GC. Prevalence of high-risk plaques and risk of stroke in patients with asymptomatic carotid stenosis: a meta-analysis. JAMA Neurol 2020;77:1524–35. 10.1001/jamaneurol.2020.2658 32744595 PMC7400201

[6] Mazurek A, Malinowski K, Rosenfield K, Capoccia L, Speziale F, de Donato G, et al Clinical outcomes of second- versus first-generation carotid stents: a systematic review and meta-analysis. J Clin Med 2022;11:4819. 10.3390/jcm11164819 36013058 PMC9409706

[7] Mazurek A, Malinowski K, Sirignano P, Kolvenbach R, Capoccia L, De Donato G, et al Carotid artery revascularization using second generation stents versus surgery: a meta-analysis of clinical outcomes. J Cardiovasc Surg 2023;64:570–82. 10.23736/S0021-9509.24.12933-3 38385840

[8] Sackett DL, Rosenberg WMC, Gray JAM, Haynes RB, Richardson WS. Evidence based medicine: what it is and what it isn’t. BMJ 1996;312:71–2. 10.1136/bmj.312.7023.71 8555924 PMC2349778

[9] Grunwald IQ, Mathias K, Bertog S, Snyder KV, Sievert H, Siddiqui A, et al World Federation for Interventional Stroke Treatment (WIST) multispecialty training guidelines for endovascular stroke intervention. Adv Interv Cardiol 2023;19:6–13. 10.5114/aic.2023.124742 PMC10114172 37090217

[10] Karpenko A, Bugurov S, Ignatenko P, Starodubtsev V, Popova I, Malinowski K, et al Randomized controlled trial of conventional versus MicroNet-covered stent in carotid artery revascularization. JACC Cardiovasc Interv 2021;14:2377–87. 10.1016/j.jcin.2021.08.005 34736737

